# Electrophysiological correlates of fearful and sad distraction on target processing in adolescents with attention deficit-hyperactivity symptoms and affective disorders

**DOI:** 10.3389/fnint.2012.00119

**Published:** 2012-12-19

**Authors:** Anthony Singhal, Andrea T. Shafer, Matthew Russell, Bridget Gibson, Lihong Wang, Sunita Vohra, Florin Dolcos

**Affiliations:** ^1^Department of Psychology, University of AlbertaEdmonton, AB, Canada; ^2^Centre for Neuroscience, University of AlbertaEdmonton, AB, Canada; ^3^Department of Psychiatry, Duke UniversityDurham, NC, USA; ^4^Department of Pediatrics, University of AlbertaEdmonton, AB, Canada; ^5^Department of Psychology, Neuroscience Program, Beckman Institute for Advanced Science and Technology, University of Illinois at Urbana-ChampaignUrbana, IL, USA

**Keywords:** ADHD, adolescents, anxiety, attention, depression, emotion, event-related potentials

## Abstract

In this study we used event-related brain potentials (ERP) as neural markers of cognitive operations to examine emotion and attentional processing in a population of high-risk adolescents with mental health problems that included attention deficit and hyperactivity disorder (ADHD), anxiety, and depression. We included a healthy control group for comparison purposes, and employed a modified version of the emotional oddball paradigm, consisting of frequent distracters (scrambled pictures), infrequent distracters (sad, fearful, and neutral pictures), and infrequent targets (circles). Participants were instructed to make a right hand button press to targets and a left hand button press to all other stimuli. EEG/ERP recordings were taken using a high-density 256-channel recording system. Behavioral data showed that for both clinical and non-clinical adolescents, reaction time (RT) was slowest in response to the fearful images. Electrophysiological data differentiated emotion and target processing between clinical and non-clinical adolescents. In the clinical group we observed a larger P100 and late positive potential (LPP) in response to fearful compared to sad or neutral pictures. There were no differences in these ERPs in the healthy sample. Emotional modulation of target processing was also identified in the clinical sample, where we observed an increase in P300 amplitude, and a larger sustained LPP in response to targets that followed emotional pictures (fear and sad) compared to targets that followed neutral pictures or other targets. There were no differences in these target ERPs for the healthy participants. Taken together, we suggest that these data provide important and novel evidence of affective and attention dysfunction in this clinical population of adolescents, and offer an example of the disruptive effects of emotional reactivity on basic cognition.

## Introduction

Emotion can both enhance and impair cognition and performance (Dolcos et al., 2011; Chan and Singhal, 2013). For instance, increased attention to emotional stimuli can also lead to distracting effects on cognitive performance if the emotional information is task-irrelevant (Dolcos and McCarthy, 2006; Shafer et al., 2012). These opposing effects of emotion are exacerbated in clinical conditions, such as depression and anxiety, where increased emotional distractibility is observed. This heightened susceptibility to emotional distraction may, in part, be due to faulty regulatory mechanisms that help individuals cope in the presence of unwanted emotional stimuli. The ability to regulate emotion is a complex phenomenon that begins to develop in infancy, and continues through the childhood and adulthood years. Moreover, a healthy set of emotional regulatory strategies are considered to be highly associated with overall positive health states and general wellbeing (Thompson and Calkins, 2006; Denkova et al., 2012). In recent years there have been important advances in the neuroscientific study of emotion and emotion regulation (see Dolcos et al., 2011). In particular, the neural basis of emotion regulation has received considerable research interest because of the compelling argument that certain types of psychopathology are linked to a fundamental *dys*regulation in emotion processing (Davidson, 2002; Phillips et al., 2008). This dysregulation has been described as involving an imbalance between basic affective processing and higher-level executive processes including top–down attentional control (Johnson et al., 2005). Moreover, in pediatric populations emotion regulation is likely of paramount importance in the development of stable and normal cognitive function over time (Lewis et al., 2006). It is widely known that psychopathologies with a childhood onset are associated with a higher incidence of relapse, heightened resistance to therapy, and other long-term varied health problems (Snyder, 2001). It has been suggested that children at risk for depression may be vulnerable to other risks due to trouble with self-regulation of their own emotions as well as receiving inconsistent regulatory management from caregivers and peers due to their reactivity (Thompson and Calkins, 2006). That is, these children may be offered less support and help with alternate strategy formation that is critical for normal development. Similar evidence exists in the attention deficit and hyperactivity disorder (ADHD) literature. Where emotional dysregulation contributes to behavioral excess, impulsive responding, and delayed cognition that ultimately leads to the child's feelings of heightened frustration and interferes with normal development of socio-emotional skills (Walcott and Landau, 2004).

From a neuroimaging point of view, emotion regulation processes have been shown to correlate with activation in dorsal–lateral, medial prefrontal, and lateral parietal cortices associated with attentional control processes, as well as changes in activation in the amygdala, ventral–lateral, and ventral medial prefrontal regions associated with emotional re-appraisal and attenuated emotional reactivity (Beauregard et al., 2001; Yamasaki et al., 2002; Urry et al., 2006). Although much of the relevant cognitive neuroscience literature in this area has been provided by fMRI research, important contributions to this field have also been made through event-related potential (ERP) methods. ERP reflect synchronous post-synaptic neural activity that is time locked to the onset of an eliciting stimulus, and are typically characterized by their peak amplitude, time-to-peak latency, and scalp topography (Luck, 2005). This technique is highly valuable for the study of human cognitive phenomena because they are non-invasive and provide a reflection of neural activity with excellent temporal resolution in the order of milliseconds (Luck, 2005). Thus, they are useful for modeling near simultaneous neuronal activity, while at the same time are highly suitable for studying brain function in pediatric and clinical populations. The primary focus of the present study was to examine the ERP markers of emotion and emotional regulation in youth suffering from affective and attentional disorders while engaged in an emotional oddball task (modified from Wang et al., 2005) that allowed for the assessment of neural activity in response to both emotional stimuli and non-emotional stimuli requiring attentional control, as well as the interactions between them.

ERP studies of emotion processing employing stimuli from the International Affective Picture System (IAPS), a standardized set of photographs that vary along dimensions such as emotional valence and arousal (Lang et al., 2005), identified specific ERP components sensitive to emotion modulation. Using IAPS stimuli, research has shown that emotional images are often associated with an increase in early and sustained attention that presumably facilitates the processing of emotional information, and is reflected by a modulation of the amplitude of the ERPs. For example, both the P100 and the late positive potential (LPP) are well characterized ERP components that are sensitive to modulations by emotion [see Olofsson et al. (2008) for a review]. The P100 is a positively deflecting waveform that typically occurs between 80 and 200 ms post-stimulus onset and has been shown to be a marker of extrastriate activity (Clark et al., 1995). The P100 is the most consistently found early component that can be modified by fearful emotion (Eimer and Holmes, 2002; Smith et al., 2003; Carretie et al., 2004; Delplanque et al., 2004; Pourtois et al., 2005; Holmes et al., 2006). While the P100 is commonly modulated by emotion, the topography of the modulation has varied from occipital, to lateral-occipital, to parietal, to frontal locations. It has been suggested that this fluctuation in topography is largely due to methodological and task effects (Olofsson et al., 2008).

The LPP is a positive deflection that peaks over parietal electrode sites at latencies that are after 300 ms, and is evident throughout the presentation duration of the eliciting emotional picture or word. It has been shown to be larger in amplitude in response to aversive stimuli compared to neutral stimuli, as well as stimuli that are highly arousing (Dolcos and Cabeza, 2002; Schupp et al., 2004; Weinberg and Hajcak, 2010). Moreover, the larger LPP effect in response to emotional stimuli is not sensitive to habituation effects associated with repeated stimulus presentation (Olofsson and Polich, 2007) as is the case of galvanic skin conductance (GSR), electromyography (EMG), and amygdala activation in fMRI (Breiter et al., 1996; Codispoti et al., 2006, 2007). The LPP appears to require the conscious awareness of the eliciting stimulus (Williams et al., 2007), and shows consistent morphology over time within subjects (Codispoti et al., 2006). In terms of its functionality, it has been argued that the LPP reflects an increase in sustained attention in order to facilitate the extended processing of motivational information, including higher cognitive processes such as memory encoding and retention (Koenig and Mecklinger, 2008). The LPP has been linked to activity in the occipital, parietal, and inferior temporal lobes (Keil et al., 2002; Sabatinelli et al., 2007), perhaps also reflecting downstream activity due to initial emotional modulation of the amygdala (Hajcak et al., 2010). Despite relatively limited research examining the LPP in children and youth, it has been shown that a measurable LPP is evident in response to emotional face presentation in populations as young as 7 month old (Leppanen et al., 2007). More recently, Hajcak and Dennis (2009) showed that the LPP is larger in response to emotional compared to neutral content in IAPS stimuli in children, and it has been suggested that children who have suffered abuse elicit larger LPP waves to stimuli that portray threatening and anger situations (Shackman et al., 2007). Moreover, it has been argued that since the LPP is a viable marker of fear-based processing, it may be useful as an indicator of emotional dysregulation in clinical populations, including pediatric affect disorders (Solomon et al., 2012).

Another ERP component that has been shown to be strongly related to attention and also emotion processing is the P300, which is observed as a large positive waveform maximal over midline central and parietal electrode sites peaking between 300 and 500 ms after stimulus onset (Sutton et al., 1965). Extensive literature supports the idea that the P300 wave has multimodal generators (Kok, 2001) and peaks once a task relevant stimulus has been evaluated. It is typically observed when attention is paid to a stimulus train which has both frequent and infrequent (oddball) trials. It has been shown that the peak latency of the P300 increases if the categorization of a target stimulus becomes more difficult suggesting it is also involved in low level perception (Kutas et al., 1977; Coles et al., 1995). There is an agreement that P300 amplitude reflects the intensity of processing (Donchin et al., 1986a,b) as well as perceptual-central resources (Donchin et al., 1986a; Kramer and Spinks, 1991) within a multiple capacity framework (Wickens, 1984; Singhal and Fowler, 2004, 2005). In a study co-registering ERP and fMRI data, the brain networks underlying the visual P300 (oddball P3b) were localized to both parietal cortex and inferior temporal cortex (Bledowski et al., 2004). It has also been long argued that the multimodal nature of P300 is likely due to significant frontal lobe contribution (Johnson, 1993). The P300 has been shown in some studies to be larger in response to affective images compared to neutral images (Carretie et al., 2004) and this effect has been attributed to the idea that emotion directs the allocation of attention and, it has been further argued that emotional stimuli are “natural targets” because of their strong salience and motivational relevance (Johnston et al., 1986; Sabatinelli et al., 2005). In the context of emotion regulation, it has been argued that the amplitude of P300 may reflect the amount of cognitive resources allocated to the processing of information that follows an emotional stimulus (Ellis and Ashbrook, 1988). Further, it has been suggested that this process may function to critically subserve regulatory processes (Deveney and Pizzagalli, 2008).

Previous research examining emotion regulation and attentional control in youth suggests that this population maybe less well equipped to properly inhibit unwanted allocation of their attentional resources toward distracting emotional information. Furthermore, youth suffering from mental health concerns including attentional and affective disorders may have more difficulty with this type of inhibition. However, to date the underlying neural mechanisms of this phenomenon have not been fully elucidated. The primary research purpose of this study was to examine the nature of these emotion and attention ERP markers (i.e., P100, LPP, and P300) in a population of youth with potential dysfunction in emotion regulation and attention because they had been diagnosed with symptoms related to affective disorders and ADHD. To that end, adolescents suffering with mental health problems and a healthy control group of participants performed a modified version of the emotional oddball paradigm (after Wang et al., 2005), that allowed for the assessment of emotion processing, goal directed attentional processing, and the interaction between the two. For distracter processing, we predicted differences in behavioral and ERP data such that reaction time (RT) would be delayed and early and late ERP components would be modified by emotional images compared to neutral distracter images. Specifically, the P100 and LPP amplitude would be enhanced by affective compared to non-affective distracters. For target processing, we predicted differences in behavioral and ERP data such that RT and P300 amplitude in response to targets would differ as a result of the preceding distracter type. Moreover, we predicted that the pattern of behavioral and neural responses for both distracters and targets would be different between our clinical and healthy control groups.

## Methods

### Participants

Twenty-seven (10 male, 2 left-handed) adolescents (12–17 years; average age = 14.3; SD = 1.27) were recruited from a residential mental-health treatment facility in the City of Edmonton, Alberta, Canada. These individuals were clinically diagnosed with DSM-IV Axis-1 disorders including ADHD combined, predominantly inattentive type and predominantly hyperactive/impulsivity type, oppositional defiant disorder, conduct disorder, depressive disorders (major depression and dysthymia), and anxiety disorders (including generalized anxiety disorder; post-traumatic stress disorder; and anxiety disorder). Clinical characteristics of these participants were summarized in Table 1. For summary purpose, we grouped depressive disorders and anxiety disorders as distress disorders. As shown in Table 1, there were pre-existing or co-occurring co-morbidities. Six healthy control adolescents were recruited from the City of Edmonton (three male, 13–16 years, average age = 14.67; SD = 1.21). All participants had normal or corrected-to-normal vision. Informed consent and assent were obtained from parental guardians and participants before participating. The experimental protocol was approved for ethical treatment of human participants by the Health Research Ethics Board at the University of Alberta. ERP data was assessed on a subset of 10 (5 male, 1 left-handed) clinically diagnosed adolescents (13–16 years; average age = 14.1 years; SD = 1.2). These 10 were chosen because they had the best ERP signal-to-noise ratio as determined by visual inspection. ERP data were assessed for all six healthy control (non-clinical) adolescents.

**Table 1:** **Diagnostic and medication information for the 27 clinical adolescents**.

**Diagnosis**	**Number (male/female)**	**Medication (number of patients)**			
		**None/unknown**	**Stimulants**	**Anti-depressants**	**Others**
**ADHD CO-MORBID WITH ONE OR MORE FOLLOWING DISORDERS**					
ODD, OCD, PCRP, SRC, RAD, IED, conduct disorder, learning disorders	10 (5/5)	3/1	5	SSRI-2	Atypical antipsychotic-2
				NRI-1	Benzodiazepine-1
					β-adrenergic receptor agonist-1
Distress disorders (one or more of the following: major depression, dysthymia, anxiety GAD, PTSD, social phobia)	4 (2/2)	1/0	2	SSRI-2	Atypical antipsychotic-1
**DISTRESS DISORDER**					
Major depression	1 (0/1)	–	–	SSRI-1	Atypical antipsychotic-1
**DISTRESS DISORDERS (MAJOR DEPRESSION, DYSTHYMIA, GAD, PTSD) CO-MORBID WITH ONE OR MORE FOLLOWING DISORDERS**					
Distress disorder	1 (1/0)	–	–	NDRI-1	Atypical antipsychotic-1
ODD, PCRP, SRC, RAD, conduct disorder, substance abuse, sexual abuse	8 (1/7)	–	2	SSRI-7	Atypical antipsychotic-4
				NDRI-2	Benzodiazepine-1
				NRI-1	
**OTHERS: TWO OR MORE FOLLOWING DISORDERS**					
ODD, PCRP, conduct disorder	3 (1/2)	3/0	–	–	–
Total	27 (10/17)	7/1	9	SSRI-12	Atypical antipsychotic-9
				NDRI-3	Benzodiazepine-2
				NRI-2	β-adrenergic receptor agonist-1

ADHD, attention-deficit/hyperactivity disorder; ODD, oppositional defiant disorder; OCD, obsessive-compulsive disorder; PCRP, parent-child relation problem; RAD, reactive attachment disorder of infancy or early childhood; SRC, sibling-relational conflict; IED, intermittent explosive disorder; SSRI, selective serotonin reuptake inhibitors; NRI, norepinephrine reuptake inhibitors; NDRI, norepinephrine-dopamine reuptake inhibitors.

### Task and stimuli

Participants performed a modified version of the emotional oddball paradigm (Wang et al., 2005) which consisted of frequent stimuli serving as the baseline [scrambled pictures, 79% (465 trials)], infrequent distracters and oddball targets, 21% (124 trials). Infrequent distracters consisted of sad and fearful pictures (13 trials each), neutral pictures (26 trials), and positive pictures (4 trials). The oddball targets (circles) were sub-grouped according to their preceding infrequent stimulus type [i.e., target-after-sad (11 trials), target-after-fear (11 trials), target-after-target (24 trials), and target-after-neutral stimuli (22 trials)]. To ensure that sad and fear pictures were paired to a neutral picture that possessed similar visual qualities (e.g., sad picture, man sitting and crying; neutral picture, another man sitting with no overt emotional expression), the neutral pictures were originally subdivided into neutral paired with sad and neutral paired with fear. However, for analyses these separate neutral categories were collapsed resulting in one neutral picture and one target-after-neutral category. Positive pictures only served as emotional anchors, to provide a context for ratings, and were not included in the analyses. The infrequent distracter stimuli (sad, fearful, and neutral pictures) were selected from IAPS based on normative ratings for valence and arousal and were supplemented with in-house pictures used in previous studies (Wang et al., 2005, 2008). Participant's ratings of the distracter categories did not differ between the clinical and non-clinical groups, *F*_(4, 80)_ = 0.3, *p* = 0.88 for valence and *F*_(4, 80)_ = 0.34, *p* = 0.85 for arousal. There was main effect of valence, *F*_(2, 80)_ = 129.55, *E* = 0.65, *p* < 0.001, and a main effect of arousal *F*_(2, 80)_ = 44.22, *E* = 0.79, *p* < 0.001. The fear images were rated as most negative (fear > sad > neutral) and most arousing (fear > sad > neural). The mean valence/arousal scores for each distracter type rated by the 27 clinical adolescents (on a scale from 1 to 9) were as follows: 5.22/2.48 for neutral; 2.65/5.22 for fear; and 2.87/4.04 for sad. The mean valence/arousal scores rated by the 10 ERP clinical adolescents were as follows: 5.34/2.32 for neutral; 2.58/4.62 for fear; and 2.83/3.42 for sad. The mean valence/arousal scores as rated by the six non-clinical adolescents were as follows: 5.3/2.21 for neutral; 2.4/5.33 for fear; and 3.05/3.97 for sad. The infrequent circle targets varied in size and color so that each target stimuli was unique. The frequent distracter stimuli (scrambled pictures) were digitally scrambled versions of the picture stimuli and thus contained the same average spatial frequency and luminance as the emotional and non-emotional pictures. Participants made one button press to all frequent (i.e., scrambled pictures) and infrequent (i.e., neutral, sad, and fear pictures) stimuli, and they made another button press to all target stimuli.

### Event-related potential (ERP) recording and analyses

ERPs were recorded using a high-density 256-channel Geodesic Sensor Net (Electrical Geodesics Inc., Eugene, OR), amplified at a gain of 1000 and recorded at a sampling rate of 250 Hz [impedance <50 KΩ and initially referenced to the vertex electrode (Cz)]. Using Netstation (Version 4.4.2, Electrical Geodesics Inc., Eugene, OR), data were bandpass filtered from 0.1 to 30 Hz, grand average re-referenced offline, and segments were constructed around events of interests from 300 ms pre-stimulus to 800 ms post-stimulus. Data were also baseline corrected (−300 to 0 ms), and corrected for eye-movement artifacts. A min of five epochs per condition were necessary for the participant to be included in ERP analyses. The individual waveforms were visually inspected, and clear components of interests (i.e., P100, P300, and LPP) were identified for each participant at or near electrodes sites shown in prior literature to display maximal amplitudes. More specifically, because our primary goal of the study is to investigate emotional dysregulation effects on cognition in a clinical population, we first investigated significant effects in the clinical group. A secondary analysis on the non-clinical group data was performed for confirmation. Thus, analyses were observation-driven with ERP inspection in the clinical group for distracter and target ERPs at cardinal electrode clusters. Significant effects that were identified in the clinical group were then compared to the corresponding electrode sites in the non-clinical control group. Mean amplitude data for late (LPP and P300) ERP components and maximum amplitude data for early (P100) ERP components were then extracted. Time windows for each component were determined from visual inspection and were 300–549 ms post-stimulus for the P300, 550–800 ms post-stimulus for LPP, and 100–200 ms post-stimulus for P100. Since data was acquired with a high-density net consisting of 256 electrodes, we also employed an extent threshold of three adjacent electrodes for all components of interests.

### Experimental procedures

**Figure 1:**
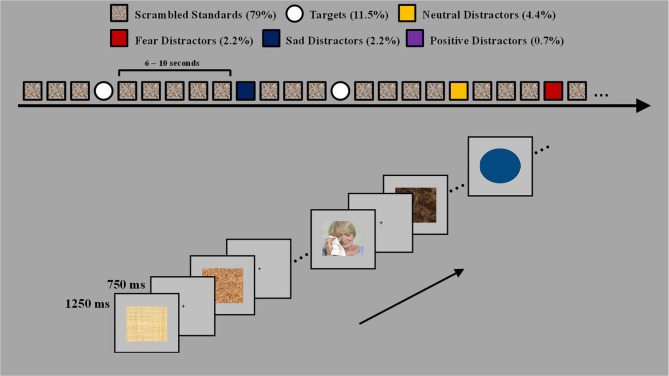
**Task design.** The task used four types of rare events, fear, sad, neutral distracters, and target circles varying in size and color. The four types of rare events were presented pseudorandomly between the standard scrambled pictures and were separated by 6–10 s. Participants were instructed to make a left hand button press to all scrambled pictures and any picture with a person and to make a right hand button press to all target stimuli.

The oddball trials (i.e., infrequent distracters and target stimuli) were divided into 4 runs of 25 trials and 1 run of 24 trials. To avoid induction of mood states, the negative distracter oddball trials within each run were pseudorandomized so that no more than two trials of the same valence type were consecutively presented. The inter-trial interval was 2 s. Each trial started with the presentation of a stimulus (frequent, infrequent distracter, or a target) presented for 750 ms and was followed by a fixation screen for 1250 ms. To prevent the participants from anticipating the occurrence of a stimulus the interval between rare stimuli (i.e., the infrequent distracters and targets) was randomized on an exponential distribution with a median of 8 s and a range between 6 and 10 s (see Figure 1). The participants' task was to indicate whether the stimulus was a target or non-target by pressing a button. Participants were instructed to make a right hand button press any time they saw a target (circle) and a left hand button press to all other stimuli (i.e., frequent scrambled and infrequent sad, fearful, neutral, and positive distracters). Participants were also instructed to respond as soon as the image was presented and to respond as quickly and as accurately as possible, and to experience any feelings and thoughts the pictures might trigger.

### Statistical analyses

For comparison of clinical and non-clinical groups, behavioral RT and ERP (P100 max amplitude; P300 and LPP mean amplitude) data in response to distracters and targets were analyzed via two separate mixed model analyses of variances (ANOVA) tests. For each ANOVA the between subject variable was group (clinical and non-clinical). For the distracter ANOVAs the within subjects variable was distracter type (neutral, fear, and sad), while for the target ANOVAs the within subject variable was target type (target-after-target, target-after-neutral, target-after-fear, and target-after-sad). These mixed model analyses were performed using the clinical group (*n* = 10) that had both behavioral and ERP data. For within group comparisons One-Way repeated measures ANOVA were performed for distracter and target data. The distracter ANOVA assessed responses to the infrequent sad, fearful, and neutral distracters. The target ANOVA assessed responses to the targets as a function of the preceding rare stimulus type (i.e., target-after-target; target-after-neutral; target-after-sad; and target-after-fear). For all analyses the *p*-value corresponding to the Greehouse-Geisser correction is reported. The epsilon values are reported only where significance was found. *Post-hoc* comparisons were performed where appropriate using the Fisher LSD test. The within group analyses were performed on all three groups (i.e., non-clinical, clinical with 10 participants, and clinical with 27 participants) separately. In the behavioral analyses trials were excluded if they were incorrect and if RT data were ≤175 ms or ≥2000 ms. While error rates were significantly greater for target (*M* = 12.4%, SE = 2.4%) compared to distracter (*M* = 4.1%, SE = 1.4%) stimuli, *F*_(1, 40)_ = 8.84, *p* = 0.005, they did not differ as a function of group, *F*_(2, 40)_ = 0.91, *p* = 0.41, nor did they differ within stimulus type (i.e., within distracter and target stimuli), *F*_(2, 80)_ = 0.93, *p* = 0.4, for distracters, and *F*_(3, 120)_ = 0.16, *p* = 0.93, for targets. For the ERP analyses, all trials were included in the analyses as the number of trials usable after data processing was low.

## Results

### Increased behavioral impact of fearful distracters

Processing of fearful distracters was associated with longer RTs in both clinical and control groups. There were no differences between clinical (*n* = 10) and non-clinical adolescents (*n* = 6) in RT to distracters, *F*_(1, 14)_ = 0.004, *p* = 0.95, or the Distracter Type × Group interaction, *F*_(2, 28)_ = 0.05, *p* = 0.86. There was a main effect of Distracter Type, *F*_(2, 28)_ = 8.35, *E* = 0.59, *p* = 0.008, and *post-hoc* comparisons using Fisher LSD test showed RT to fear distracters was significantly longer than to neutral, *p* = 0.006, and sad, *p* = 0.01 distracters, where the later two type of distracters were not different from each another, *p* = 0.48. Assessing the effect of Distracter Type on RT for each group separately showed that the same pattern was present for both clinical, *F*_(2, 18)_ = 4.11, *E* = 0.58, *p* = 0.065, and non-clinical, *F*_(2, 10)_ = 9.18, *E* = 0.66, *p* = 0.017, adolescents. Similar to the above results for the clinical sub-sample of 10 and the non-clinical sample of six, the analysis on RT data for all 27 participants also showed a main effect of Distracter Type, *F*_(2, 52)_ = 7.57, *E* = 0.071, *p* = 0.004. *Post-hoc* comparisons using Fisher LSD test showed longer RT to the fearful distracters than to neutral (*p* = 0.002) or sad (*p* = 0.009) distracters, but the latter two were not significantly different from each another (*p* = 0.45) (see Table 2 for mean and standard error RT data for each distracter category for clinical samples of 27 and 10 and the non-clinical sample of six).

**Table 2:** **Mean reaction time (RT) and standard error (SE) data to distracters and targets for both the large sample of 27 participants and the small sample of 10 participants**.

**Distracter type**	**Group**	**Neutral**	**Fear**	**Sad**	
RT (SE)	Clinical *n* = 27	578.49 (24.63)	629.43 (32.48)	568.59 (23.18)	
	Clinical *n* = 10	620.65 (47.08)	699.54 (64.24)	619.77 (47.92)	
	Non-clinical *n* = 6	633.38 (70.17)	704.65 (57.07)	617.95 (65.38)	
**Target type**	**Group**	**Target-after-neutral**	**Target-after-fear**	**Target-after-sad**	**Target-after-target**
RT (SE)	Clinical *n* = 27	527.91 (16.02)	536.98 (17.89)	538.31 (18.27)	526.45 (15.85)
	Clinical *n* = 10	555.94 (26.18)	557.61 (25.19)	548.62 (31.04)	544.5 (24.15)
	Non-clinical *n* = 6	502.81 (31.07)	499.11 (26.01)	515.9 (20.87)	498.88 (24.76)

### ERP evidence of increased processing of fearful distracters in clinical adolescents

**Figure 2:**
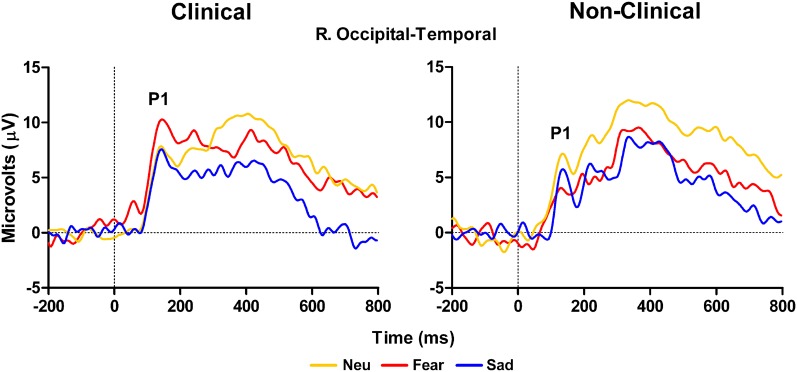
**Grand average waveforms in response to distracter stimuli over right temporal-occipital electrodes showing larger peak P100 amplitude to fearful distracters compared to neutral and sad distracters for clinical adolescents (left panel) compared to non-clinical adolescents (right panel).** Neu, Neutral pictures; Fear, Fear Pictures; Sad, Sad Pictures.

The ERP data revealed an impact of Distracter Type and Group for both early (P100) and late (LPP) components in response to the distracter images. First, the P100 amplitude at right hemisphere occipital-temporal electrodes (P10 in 10–10 topography) showed a significant interaction between Distracter Type and Group, *F*_(2, 28)_ = 4.41, *E* = 0.88, *p* = 0.027, but no main effect of Distracter Type [*F*_(2, 28)_ = 0.92, *p* = 0.4] or Group [*F*_(1, 14)_ = 2, *p* = 0.2] effect. There was a main effect of Distracter Type for the clinical sample, *F*_(2, 18)_ = 3.83, *E* = 0.91, *p* = 0.047, where replicating the observed behavioral pattern, *post-hoc* comparisons using Fisher LSD test showed overall the amplitude was larger for fearful images relative to both neutral (*p* = 0.02) and sad (*p* = 0.05), where the later two were not different from each another (*p* = 0.82), see Figure 2, left panel. Whereas, for the non-clinical sample there was no effect of Distracter Type on P100 amplitude, *F*_(2, 10)_ = 1.99, *p* = 0.2, see Figure 2, right panel, and Table 3.

**Table 3:** **Mean ERP amplitudes and standard error (SE) for the LPP, P100, and P300**.

**ERP component**	**Electrode cluster**	**Group**	**Distracter type**		
			**Neutral**	**Fear**	**Sad**	
P100	R. occipital-temporal	Clinical *n* = 10	8.76 (0.79)	11.37 (1.33)	8.48 (1.25)	
		Non-clinical *n* = 6	9.03 (1.29)	6.22 (1.5)	7.02 (1.42)	
LPP	Parietal	Clinical *n* = 10	3.36 (0.98)	6.34 (1.18)	3.92 (0.95)	
		Non-clinical *n* = 6	6.34 (1.26)	6.8 (1.52)	5.76 (1.22)	
	L. temporal	Clinical *n* = 10	1.02 (1.06)	3.75 (1.09)	−0.66 (0.87)	
		Non-clinical *n* = 6	3.1 (1.37)	5.49 (1.42)	2.11 (1.13)	
			**Target type**			
			**Target-after-neutral**	**Target-after-fear**	**Target-after-sad**	**Target-after-target**
P300	L. parietal	Clinical *n* = 10	4.12 (1.19)	5.68 (0.97)	5.5 (1.21)	3.23 (1.31)
		Non-clinical *n* = 6	4.44 (1.54)	5.5 (1.26)	5.2 (1.56)	5.71 (1.69)
LPP	L. occipital-temporal	Clinical *n* = 10	−0.81 (0.99)	1.85 (0.76)	2.04 (0.99)	−0.75 (0.82)
		Non-clinical *n* = 6	2.29 (1.28)	3.37 (0.98)	2.03 (1.28)	2.03 (1.28)

**Figure 3:**
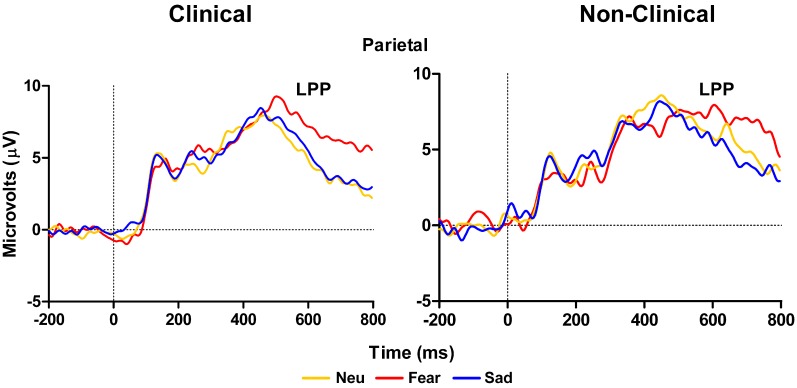
**Grand average waveforms in response to distracter stimuli.** The late positive potential (LPP) over left, midline, and right parietal electrodes is larger for fearful distracters compared to neutral and sad distracters for clinical adolescents (left panel), but not non-clinical adolescents (right panel). Neu, Neutral pictures; Fear, Fear Pictures; Sad, Sad Pictures.

There was no Distracter Type × Group interaction effect for the LPP at the left, midline, or right parietal electrodes (P3, Pz, and P4 in 10–10 topography), *F*_(2, 28)_ = 1.78, *p* = 0.19, nor was there a main effect of Group, *F*_(1, 14)_ = 1.17, *p* = 0.3. However, there was a significant effect of Distracter Type, *F*_(2, 28)_ = 5.1, *E* = 0.81, *p* = 0.02. Analyses examining the effect of Distracter Type on parietal LPP amplitude for clinical and non-clinical samples separately found a main effect of Distracter Type for clinical, *F*_(2, 18)_ = 9.52, *E* = 0.94, *p* = 0.002, but not non-clinical adolescents, *F*_(2, 10)_ = 0.48, *p* = 0.55, see Figure 3. *Post-hoc* comparisons for the clinical data using Fisher LSD test identified a pattern similar to the behavioral and P100 data with this main effect driven by larger mean amplitude in response to fearful distracters compared to neutral (*p* = 0.001) and sad (*p* = 0.01) distracters, again there were no difference between sad and neutral distracters (*p* = 0.69), see Figure 3, left panel, and Table 3. There was no effect of Distracter Type, *F*_(2, 28)_ = 0.12, *p* = 0.84, Group, *F*_(1, 14)_ = 0, *p* = 1, or Distracter Type × Group interaction, *F*_(2, 28)_ = 0.5, *p* = 0.57, on P300 amplitude measured at parietal electrodes.

**Figure 4:**
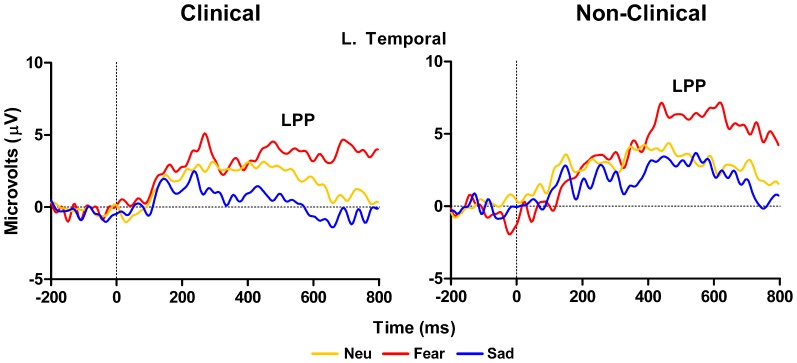
**Grand average waveforms from left temporal electrodes showing a larger LPP for clinical adolescents in response to high arousal negative fearful distracters compared to neutral distracters.** The LPP did not differ between neutral and sad distracters (left panel). Non-clinical adolescents had no significant differences between distracter groups (right panel). Neu, Neutral pictures; Fear, Fear Pictures; Sad, Sad Pictures.

Left temporal electrodes (TP7 in 10–10 topography) showed a main effect of Distracter Type, *F*_(2, 28)_ = 10.57, *E* = 0.87, *p* = 0.001, but no main effect of Group, *F*_(2, 14)_ = 2.75, *p* = 0.12, or Distracter Type × Group interaction, *F*_(2, 28)_ = 0.19, *p* = 0.8. Pairwise comparison using Fisher LSD test showed LPP mean amplitude was larger for fear compared to neutral distracters (*p* = 0.009) and the neutral distracters mean amplitude was larger compared to sad distracters (*p* = 0.08) (i.e., fear > neutral > sad). Investigation of the LPP for clinical and non-clinical samples separately revealed a main effect of Distracter Type for the clinical sample, *F*_(2, 18)_ = 9.21, *E* = 0.96, *p* = 0.002, and a trend effect for the non-clinical sample, *F*_(2, 10)_ = 3.08, *E* = 0.58, *p* = 0.09, see Figure 4. *Post-hoc* comparisons using Fisher LSD test showed for the clinical group the amplitude to fear distracters was larger than to neutral distracters (*p* = 0.02), which were no different from the amplitude to the sad distracters (*p* = 0.14) (i.e., fear > neutral = sad). *Post-hoc* comparisons for the non-clinical samples showed no significant differences between distracter types, even though the pattern was in the same direction as for the non-clinical sample (see Table 3).

### ERP evidence for modulation of target processing by emotional distraction in clinical adolescents

For RT data, there were no significant effects of Target Type, *F*_(3, 42)_ = 0.7, *p* = 0.51, or Group, *F*_(1, 14)_ = 1.56, *p* = 0.23, or an interaction between Target Type and Group, *F*_(3, 42)_ = 0.25, *p* = 0.8. Neither the clinical or non-clinical samples showed a main effect of Target Type on RT, *F*_(3, 27)_ = 0.44, *p* = 0.66 and *F*_(3, 15)_ = 0.54, *p* = 0.54, respectively. Analysis on the larger clinical sample using all 27 participants also did not show a main effect of Target Type on RT data, *F*_(3, 78)_ = 1.38, *p* = 0.26, although the larger analysis showed a trend level effect of sad distracter images on performance, where targets-after-sad had slower response times compared to targets-after-targets, *t*_(26)_ = 1.89, *p* = 0.07. See Table 2 for mean and standard error RT data for each target category.

**Figure 5:**
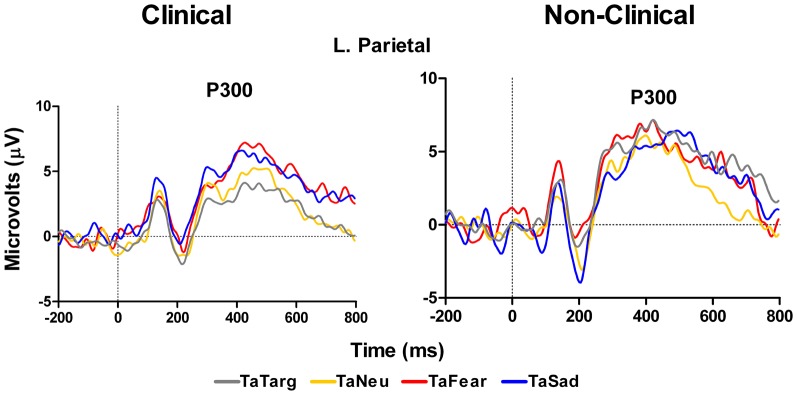
**Grand average waveforms in response to target stimuli over left hemisphere parietal electrodes.** Figure shows increase in P300 amplitude in response to targets-after-sad and targets-after-fear compared to targets-after-targets for the clinical group (left panel), but not in the non-clinical group (right panel). TaTarg, target-after-target; TaNeu, target-after-neutral; TaFear, target-after-fear; TaSad, target-after-sad.

ERP data for the P300 at left parietal electrodes (P5 and P3 in 10–10 topography) showed no main effect of Target Type, *F*_(3, 42)_ = 1.8, *p* = 0.18, or Group, *F*_(1, 14)_ = 0.11, *p* = 0.74 nor a significant interaction between Target Type and Group, *F*_(2, 42)_ = 1.8, *p* = 0.18. While this overall model was not significant, examination of Target Type for clinical and non-clinical samples separately, showed a marginal effect of Target Type for the clinical sample, *F*_(3, 27)_ = 2.86, *E* = 0.67, *p* = 0.08, but not for the non-clinical sample, *F*_(3, 15)_ = 1.68, *p* = 0.24. For the clinical group, *post-hoc* comparisons using Fisher LSD test showed that P300 amplitude to target-after-sad was larger than target-after-target (*p* = 0.03), while the amplitude to target-after-fear was marginally larger than to target-after-target (*p* = 0.07), see Figure 5 and Table 3.

**Figure 6:**
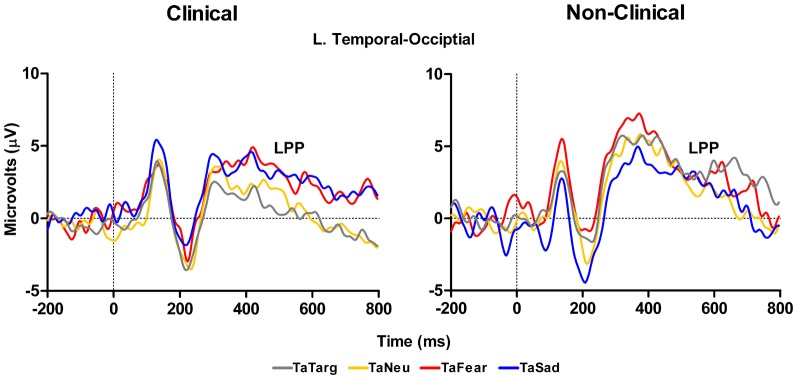
**Grand average waveforms to target stimuli from left-hemisphere temporal-occipital electrodes show an effect of valence on the LPP in response to target processing with both targets-after-sad and -fear having an increased amplitude compared to targets-after-targets for the clinical (left panel), but not non-clinical (right panel) group.** TaTarg, target-after-target; TaNeu, target-after-neutral; TaFear, target-after-fear; TaSad, target-after-sad.

In addition to the P300 results reported above, a main effect of Group, *F*_(1, 14)_ = 4.48, *p* = 0.05, with the clinical group having overall smaller amplitudes compared to the non-clinical group, and a marginal Target Type × Group interaction, *F*_(3, 42)_ = 2.78, *E* = 0.74, *p* = 0.07, was identified for LPP mean amplitude over left hemisphere temporal-occipital electrodes (TP7, P7, and P07 in 10–10 topography), see Figure 6 and Table 3. There was no main effect of Target Type, *F*_(3, 42)_ = 1.92, *p* = 0.16. To determine the effects driving the interaction, separate ANOVAs were performed on the clinical and non-clinical groups. There was a main effect of Target Type for the clinical group, *F*_(3, 27)_ = 3.69, *E* = 0.7, *p* = 0.04, but not the non-clinical group, *F*_(3, 15)_ = 2.09, *p* = 0.18. Further investigation of the main effect of Target Type in the clinical group using Fisher LSD tests showed target-after-fear and target-after-sad mean amplitudes to be larger than target-after-target, *p* = 0.05 and *p* = 0.02, respectively.

## Discussion

The main purpose of this study was to examine the morphology of ERP markers of emotion and attention in response to stimuli presented in an emotional oddball task with a group of youth primarily suffering from disorders of attention and emotion regulation. Analyses were performed on three sets of data: two from clinical samples (with or without ERP data), and one from the control sample. The task employed allowed for the comparison of behavioral and ERP responses to distracter pictures that were fearful, sad, or neutral, as well as target stimuli that were circles, which contained no emotional content. We performed analyses on the distracter events themselves (all picture types) as well as the target events that immediately followed distracters (fearful, sad, and neutral) or other targets. Our study yielded three main findings. First, we identified an increased impact of fearful distracters on behavioral performance and this difference was found for both clinical and non-clinical samples. Second, in clinical adolescents, this behavioral difference corresponded to an increase in the amplitude of early and late emotion ERP components in response to fearful relative to neutral distracters. Lastly, clinical adolescents exhibited difference in ERP morphology to targets following emotional distraction.

### Increased behavioral impact of fearful distracters

The behavioral finding in our study was that we observed longer RT in response to the fearful distracters compared to sad or neutral distracter images. We observed this fearful effect in all three of our samples, the large group of 27 participants the smaller group of 10 participants and the control group of 6 participants. This suggests that from a behavioral perspective, all three of our adolescent groups were similar and that our smaller subset clinical group is representative of the larger clinical cohort in our study. These findings show that all our participants were spending a longer time in the preparation and execution of a manual response to the fearful pictures. This delay can be interpreted as reflecting an increase in the capture of attention by the fearful images compared to sad or neutral (Ohman et al., 2001), even though they were not the main target stimuli in the task (Vuilleumier and Schwartz, 2001). Thus, the fearful images may have competed more for attention-related resources, which led to impaired performance (Dolcos and McCarthy, 2006; Zanto and Gazzaley, 2009; Denkova et al., 2010). The error rates were equivalent across all conditions, and so the differences in RT that we observed are not due to simple speed-accuracy trade-off effects. We also observed a trend in the target RT data for the large group of 27 clinical participants, where responses to targets that followed sad images were slightly slower than responses to targets that followed other targets. We are cautious to interpret this effect because our control sample is very small in comparison to the larger clinical sample, but in light of the P300 differences (discussed below), these findings are consistent with a carry-over-effect of the emotion from the affective pictures on the perception and decision making processes required for target response.

### ERP evidence of increased processing of fearful distracters in clinical adolescents

In response to the distracter pictures, we observed early and late effects in the P100 and LPP waveforms, respectively. In the case of the P100 we unexpectedly observed larger amplitudes in response to the fearful images compared to the other image types at right hemisphere occipital-temporal electrodes. Importantly, this effect was only observed in the clinical sample, and was not present in the healthy control sample. It is well-known that the P100 reflects early spatial attention operations associated with activity in extra-striate brain regions (Martinez et al., 1999), and it is one of the earliest endogenous ERP components that is sensitive to top–down control mechanisms. Thus, on the face of it this pattern of data suggests that the clinical group participants were likely allocating more attention-based resources toward images that were fearful in nature compared to the other image types. Moreover, the healthy control sample did not show evidence of this attentional strategy.

In the case of the LPP at parietal and temporal electrodes we observed larger amplitudes in response to the fearful images compared to the other two image types in the clinical sample. This effect is consistent with the RT data in response to the fearful images. Again, as with the P100 results, this pattern of data was absent in the control sample data, as there were no differences in LPP amplitude across the image types in the healthy control group; also, this effect is inconsistent with the behavioral data. The LPP has been shown to be sensitive to the arousal level of eliciting pictures (Schupp et al., 2004) and this effect appears to be verified in our data. Our ratings clearly show that the fearful images were also the most arousing. Moreover, our fear-based LPP result in the clinical sample may reflect the conscious awareness and salience of the images (Williams et al., 2007) that results from downstream processing of emotional information perhaps associated with amygdala activity (Bradley et al., 2003). Taken together with research showing that LPP amplitude correlates with anxiety level in healthy adults (MacNamara et al., 2011) and in youth with anxious attachment styles (Zilber et al., 2007), we may have observed a unique signature of anxiety and arousal associated with fear processing in our clinical population of adolescents. That is, the salience of the fear images is perceptually and cognitively heightened in our special population possibly due to a pre-existing susceptibility for fear-based reactivity.

Critically, this pattern of data was not present in the healthy control sample, which further supports our argument that our clinical adolescent group has a unique processing style for emotional information. This is particularly evident for the fear-based stimuli. Also, in the case of the healthy sample, the ERP data did not follow the behavioral data as it did in the clinical data. This finding may be explained by the small sample size of our healthy control group that makes it difficult to identify reliable physiological differences in distracter processing between groups. It could also be due to individual differences in processing of fear-based stimuli in non-clinical individuals. One other possibility is that the unique mechanisms in fear processing we observed are not intimately linked with behavior in our task. Rather, the ERP effects may reflect processes unrelated to the conscious awareness of the stimulus that are reflected in the response selection and execution process. When the LPP data is considered in conjunction with our P100 data in response to the distracter images in the clinical sample, our ERP data may be a reflection of very early attention modulation in our clinical youth population that is associated with a heightened focus toward the fearful images. The P100 is known to have neural generator sources in similar occipital-temporal regions that also underlie the LPP generation (Bradley et al., 2003), and the similar fear-based effects we observed in these two waveforms may have been facilitated by a common neural substrate related to projections between sensory and affective brain regions in our special clinical sample.

### ERP evidence for modulation of target processing by emotional distraction in clinical adolescents

The ERP in response to the target stimuli that we analyzed were the P300 and the LPP. The P300 is a well-known marker of selective attention, perceptual processes, and working memory processes (Kok, 2001). In our clinical sample at left parietal sites, the P300 was larger to targets that followed sad images and slightly larger to targets that followed fearful images compared to when targets followed other targets. These differences were absent in the control sample data. Thus we observed a target processing effect related to the preceding emotional stimuli that presumably was related to some carryover effect. This does not follow the behavioral data that showed no differences in RTs to targets following emotional images compared to targets that followed other targets. However, the P300 measure appears to be more sensitive to these putative carry-over effects than behavior, perhaps because P300 reflects perceptual processes rather than response-related processes (Kok, 2001), whereas the RT measures must reflect all operations that are engaged between stimulus presentation and response execution. Finally, the LPP data in response to targets showed a strong effect over left temporal sites where amplitudes were larger for targets following both fearful and sad images compared to targets that followed other targets. Again, this finding was only for the clinical sample, and this finding follows the P300 result over left parietal sites and may be a unique reflection of the sustained emotion processing that occurred and affected the target-related processes reflected by the P300. Thus, whereas P300 may reflect the increase in attention-related resources toward a stimulus following an emotional image in our clinical population, the LPP effect in response to targets may be more of a reflection of the sustained duration of the neural representation of the emotion itself (Hajcak et al., 2010). That is, a sustained downstream reflection of lower level processes in the amygdala and other affective structures. This sustained activity may result from the inability to disengage from processing emotional information triggered by the distracters (e.g., recollection of negative memories cued by the negative pictures), which continues after the cues disappear and affect the ability to focus on the following targets. This is consistent with mood congruent effects of emotion on memory and may be linked to emotion dysregulation as in the case of post-traumatic stress disorder (McFarlane, 2010). Importantly, this pattern of effects was absent in the control sample.

#### Caveats

Although the findings presented shed light on emotion-attention interactions in clinical compared to non-clinical adolescents, the present investigation also has limitations. First, the sample size in both the clinical and non-clinical ERP groups was relatively small. It should be under consideration that with a large sample these effects may slightly change. For example, a common finding in the emotion ERP literature is an enhanced LPP to high arousing emotional relative to neutral stimuli and even though we did not replicate this finding in our healthy control group, we clearly see a trend toward a significant LPP to fear stimuli (see Figures 3 and 4). However, in light of this, our findings are consistent with an exacerbated LPP response to high arousing emotional stimuli in clinical compared to non-clinical populations (Hajcak and Dennis, 2009). Furthermore, despite differences in the size of the behavioral only (*N* = 27), behavioral and ERP (*N* = 10), and control (*N* = 6) samples, the pattern of behavior was equivalent between all groups. We also acknowledge that our criterion of a min of five ERP trials per condition is low. A second limitation is co-morbid nature of the diagnoses in our clinical group. In fact, only one adolescent had been diagnosed with a single mental health disorder, and all others presented with two, sometimes three different disorders. Most undoubtedly the underlying neural mechanisms of these varied diagnoses differ from one another, however, and as shown here there may be some overarching abnormalities in processing that can be identified using electrophysiological measures. A third limitation of the study is the varied medication of the clinical adolescents and within our sample it is impossible to rule out the effects of medication on behavioral and ERP performance. It is possible that the behavioral measures in task performance were insensitive to group differences and those group differences observed with ERP measures were mitigated by the effects of these medications. Future studies using a similar experimental design and a larger number of subjects should further investigate these issues.

## Conclusion

Our small scale but complex study is unique in that it has examined both behavioral and ERP responses to stimuli in an emotional oddball task with a sensitive population of adolescents suffering from Axis-1 disorders including ADHD, anxiety, and depression. Moreover, we included a small sample of healthy controls individuals for comparison purposes. Overall we observed an interesting pattern of behavioral (RT) and neural responses (P100, LPP, and P300) that showed similarities (i.e., behavioral data) and differences (i.e., ERP data) in emotion and attentional processing between clinical and non-clinical samples. Fearful images impacted behavioral performance for both clinical and non-clinical samples, showing a consistent behavioral effect of fearful emotion regardless of potential underlying alterations in the neural mechanisms of emotion processing between groups. Early (P100) and late (LPP) ERP components assessing emotion processing differentiated between groups as clinical adolescents showed augmented amplitudes to fearful relative to sad and neutral pictures. Furthermore, emotion modulation of attentional processing (P300) and a sustained emotion effect on target processing (LPP) were identified for the clinical sample only. Suggesting attentional control processes in our sample of clinical adolescents were more susceptible to emotion modulation through either an increase in the initial engagement of resources or the inability to disengage from the emotional information. Taken together, these data may reflect a pattern of emotion dysregulation in adolescents suffering from Axis-1 disorders that modulates certain aspects of emotion-attention interactions. These effects did not uniquely follow the behavioral responses and perhaps reflect emotion and cognition processes that are not part of the response selection and execution process. Moreover, our results provide an example of the impairing effects that emotion and emotional reactivity can have on very basic cognitive function in sensitive individuals, but not in more robustly healthy persons. Thus, we have provided a small window into potential dysfunction between emotion and cognition in this youth population with clinical disorders.

### Conflict of interest statement

The authors declare that the research was conducted in the absence of any commercial or financial relationships that could be construed as a potential conflict of interest.
